# Prognostic Role of MELD-Lactate in Cirrhotic Patients' Short- and Long-Term Prognosis, Stratified by Causes of Cirrhosis

**DOI:** 10.1155/2022/8449579

**Published:** 2022-03-29

**Authors:** Xiao-Fu Chen

**Affiliations:** Department of Gastroenterology and Hepatology, The First Affiliated Hospital of Wenzhou Medical University, Wenzhou 325000, China

## Abstract

**Objectives:**

Recently, model for end-stage liver disease-lactate (MELD-LA) proved to be a superior predicting factor of inpatient mortality in patients with chronic liver disease. The study's objective was to evaluate the ability of MELD-LA to predict both short- and long-term mortality in critically ill cirrhotic patients stratified by causes of cirrhosis.

**Materials and Methods:**

This was a retrospective observational research of 469 cirrhotic patients entering intensive care unit. Clinical parameters and prognostic scores were measured and collected in the first 24 hours after entering intensive care unit. Follow-up duration was at least 5 years. Independent relationship between MELD-LA and mortality was evaluated by multivariate logistic regression analyses. Discrimination of scoring system was evaluated by the area under the receiver operating characteristic curve. Calibration of the score was evaluated by Hosmer-Lemeshow goodness of fit test for significance.

**Results:**

The MELD-LA score (odds ratio: 1.179, 95% confidence interval: 1.112–1.250, *P* < 0.001) was an independent risk factor for 15-day mortality. The area under the curve of MELD-LA was the highest (0.808, 95% confidence interval: 0.765–0.852) in predicting 15-day mortality and it had superior calibration. We found MELD-LA showed the best discrimination ability in cirrhotic patients caused by both alcohol and hepatitis (0.783, 95% confidence interval: 0.651–0.915) or alcohol alone (0.805, 95% confidence interval: 0.743–0.867).

**Conclusions:**

MELD-LA performs better for predicting short-term prognosis in critically ill cirrhotic patients, especially caused by both alcohol and hepatitis or alcohol alone.

## 1. Introduction

Chronic liver diseases can finally develop into liver cirrhosis and the process is irreversible [1]. Long-term liver fibrosis can lead to severe liver dysfunction and portal hypertension [2]. Patients with decompensated cirrhosis are usually accompanied by single or multiple organ failure [3–5]. The health burden of cirrhosis is increasing worldwide [6]. In compensated cirrhotic patients, unstable clinical conditions are often caused by life-threatening complications such as variceal bleeding, hepatic encephalopathy, hepato-renal syndrome, ascites, infection, and sepsis, which require entering an intensive care unit (ICU) [7, 8]. Mortality rates for cirrhotic patients in ICUs can range from 34% to 86%. Therefore, in order to select the most appropriate treatment as soon as possible and improve critically ill patients' prognosis, there is urgent need to find simple and practical forecasting methods that may help evaluate the severity of cirrhotic patients.

Model for end-stage liver disease-lactate (MELD-LA), a recently-developed clinical score, is a much better predicting factor of in-hospital mortality in cirrhotic patients than MELD alone [9]. MELD-LA performs significantly better compared with MELD for predicting in-hospital mortality in patients hospitalized for infection [10]. However, little was known about the ability of MELD-LA to predict short- and long-term prognosis in critically ill cirrhotic patients. As previously described, alcohol may be a coetiology in patients with viral (HBV and HCV)-related chronic liver diseases and ethanol intake is an independent predictor of cirrhosis in subjects with a chronic viral infection and an independent predictor of death in subjects with either HCV or HBV infection [11]. But, it is unclear if there are classes of cirrhotic patients with different causes (e.g., alcohol and hepatitis) where MELD-LA may be more useful or less. To address these problems, we aimed to evaluate the value of the MELD-LA score in predicting critically ill cirrhotic patients' short- and long-term mortality, stratified by causes of cirrhosis, in comparison to chronic liver failure-sequential organ failure assessment (CLIF-SOFA) [12], SOFA [13], Child-Pugh system [14], and MELD score [15].

## 2. Materials and Methods

### 2.1. Study Population

Patient dataset was extracted from the Medical Information Mart for Intensive Care III, an open-access database [16]. We completed “Protecting Human Research Participants” which was the National Institute of Health's training course (certificate number: 36072928) and then obtained access to the database. The database had been approved to establish by the institutional review boards of the Massachusetts Institute of Technology, in which all information associated with patients was anonymous, and this study we conducted was a retrospective observational research. Thus, ethics committee approval and informed consent were not necessary. We selected patients aged at least 18 years and stayed more than 24 h. Diagnostic criteria for cirrhosis were as follows: (i) clinical symptoms such as jaundice and ascites, (ii) laboratory test results such as prothrombin time prolongation and albumin reduction, (iii) imaging features, and (iv) histopathology [17, 18]. Patients were excluded for the following reasons: (i) malignancy, (ii) previous liver transplantation surgery, (iii) human immunodeficiency virus infection, and (iv) total bilirubin, international normalized ratio, lactate, or creatinine data lost. The study population included 469 patients after applying exclusion criteria.

### 2.2. Data Collection

Clinical parameters were collected including demographic information, vital signs, and laboratory parameters such as glucose, international normalized ratio, hemoglobin, prothrombin time, platelet count, arterial blood lactate, total bilirubin, albumin, and creatinine. The calculations of scoring systems including CLIF-SOFA, MELD, SOFA, Child-Pugh, and MELD-LA scores used the mean value of each clinical parameter within the first 24 h after entering ICU, using the formulae published [12–15]. The MELD-LA score was calculated as follows: 0.251 + 5.5257 × sqrt (lactate) + 0.338 × MELD [9]. Follow-up began the day entering ICU and lasted for at least 5 years. 15-day and 5-year all-cause mortality were main outcomes.

### 2.3. Statistical Analysis

Quantitative variables were presented median (interquartile range (IQR)) and compared by Mann-Whitney *U* test. Categorical variables were expressed absolute numbers (frequencies) and compared by chi-square test or Fisher's exact test. Independent relationship between parameters and mortality was assessed by multivariate logistic regression analyses. Odds ratio (OR) was reported with 95% confidence interval (CI). Calibration of the score was assessed by Hosmer-Lemeshow goodness of fit test for significance (*p* > 0.05). Discrimination of the score was assessed by the area under the receiver operating characteristic curve. DeLong test was used to perform the comparison between area under the curve (AUC) [19]. Sensitivity and specificity at the optimal cut-off value were compared in various scoring systems, and we stratified patients into three groups (alcoholic and hepatitis; alcoholic; alcoholic) by causes of cirrhosis. Besides, patients included were regrouped into two classes (relatively low and high risk) by MELD-LA score's optimal cut-off value. All the tests were two sided. *P* value <0.05 indicated statistical significance. All statistical analyses used STATA (version 14.0; StataCorp, State of Texas, USA).

## 3. Results

### 3.1. Baseline Characteristics

Our study included a total of 469 patients. The majority of the study population were male and white. Alcohol was the primary cause of cirrhosis. There were three patients that had three causes and 60 patients that had two. The median age of all the participants was 55.6 years. The most common causes of ICU hospitalization were infection and sepsis. The survivors and nonsurvivors were not significantly different in glucose, age, body mass index, sex, ethnicity, causes of cirrhosis, comorbidity, albumin, and length of ICU stay (*P* ≥ 0.05). Nonsurvivors had higher scores than survivors in MELD-LA, CLIF-SOFA, SOFA, MELD, and Child-Pugh scores (all *P* < 0.001). The details of the patients' baseline characteristics in both survivor and nonsurvivor cohorts are presented in Table 1.

### 3.2. Model Performance for 15-Day and 5-Year Mortality

**Figure 1:**
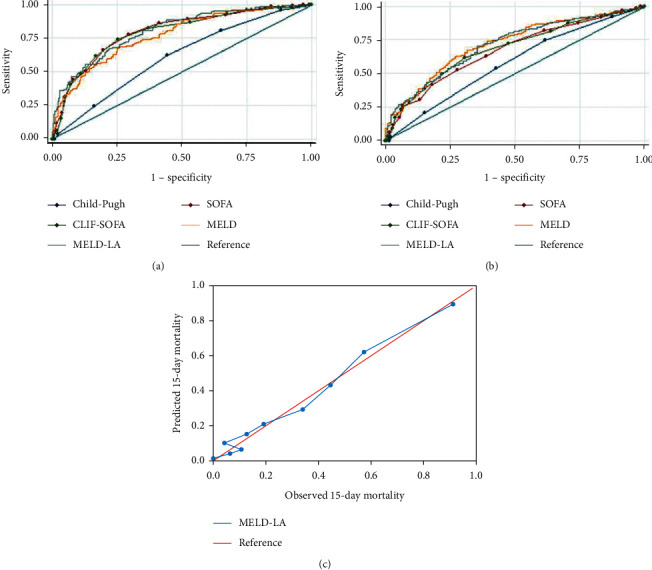
Receiver operating characteristic curves of the scoring systems for (a) 15-day and (b) 5-year mortality and (c) calibration curve of the MELD-LA score for 15-day mortality in the whole study population. SOFA, sequential organ failure assessment; CLIF-SOFA, chronic liver failure-sequential organ failure assessment; MELD, model for end-stage liver disease; MELD-LA, model for end-stage liver disease-lactate.

**Figure 2:**
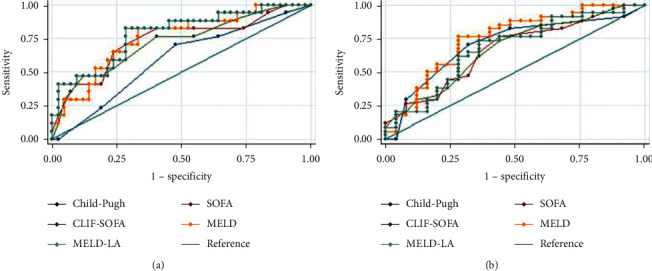
Receiver operating characteristic curves of the scoring systems for (a) 15-day and (b) 5-year mortality in critically ill patients with cirrhosis caused by both alcohol and hepatitis. SOFA, sequential organ failure assessment; CLIF-SOFA, chronic liver failure-sequential organ failure assessment; MELD, model for end-stage liver disease; MELD-LA, model for end-stage liver disease-lactate.

**Figure 3:**
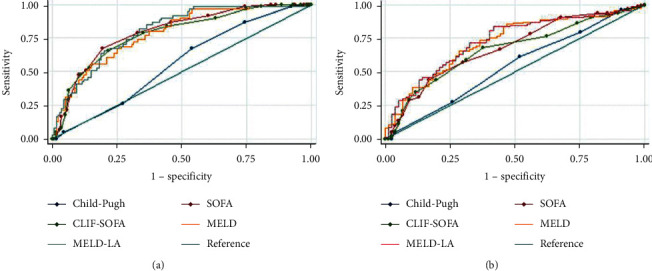
Receiver operating characteristic curves of the scoring systems for (a) 15-day and (b) 5-year mortality in critically ill patients with cirrhosis caused by alcohol alone. SOFA, sequential organ failure assessment; CLIF-SOFA, chronic liver failure-sequential organ failure assessment; MELD, model for end-stage liver disease; MELD-LA, model for end-stage liver disease-lactate.

**Figure 4:**
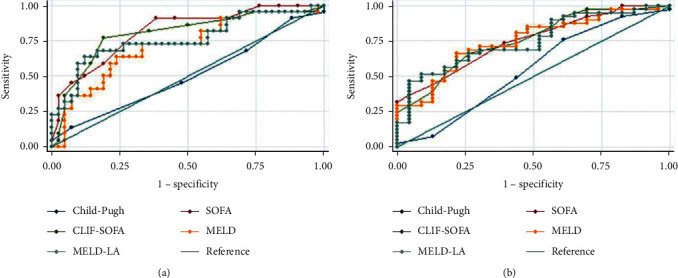
Receiver operating characteristic curves of the scoring systems for (a) 15-day and (b) 5-year mortality in critically ill patients with cirrhosis caused by hepatitis alone. SOFA, sequential organ failure assessment; CLIF-SOFA, chronic liver failure-sequential organ failure assessment; MELD, model for end-stage liver disease; MELD-LA, model for end-stage liver disease-lactate.

MELD-LA (OR: 1.179, 95% CI: 1.112–1.250), temperature (OR: 0.493, 95% CI: 0.316–0.771), respiratory rate (OR: 1.091, 95% CI: 1.025–1.161), length of ICU stay (OR: 0.715, 95% CI: 0.620–0.826), white blood cell (OR: 1.051, 95% CI: 1.010–1.094), platelet (OR: 0.994, 95% CI: 0.989–0.998), partial thromboplastin time (OR: 1.024, 95% CI: 1.007–1.041), and mechanical ventilation duration (OR: 1.012, 95% CI: 1.005–1.018) were identified as independent risk factors for 15-day mortality according to multivariate logistic regression analyses results (all *P* < 0.05). In the whole study population, as Figure 1(a) presented, the MELD-LA score performed the best in predicting 15-day mortality. When the optimal cut-off value of 15 for MELD-LA was used to predict 15-day mortality, the sensitivity and specificity were 0.81and 0.65. The 15-day, 90-day, 1-year, 3-year, and 5-year death rates for patients with low risk (the MELD-LA score <15) were 11.3% (28/248), 24.2% (60/248), 33.5% (83/248), 44.4% (110/248), and 48.4% (120/248), respectively, and for patients with high risk (the MELD-LA score ≥15), 46.6% (103/221), 63.3% (140/221), 67.9% (150/221), 73.8% (163/221), and 75.1% (166/221), respectively (all *P* < 0.001). Once the MELD-LA scores were ≥15, the risk of mortality increased significantly. As Figure 1(b) presented, for predicting 5-year mortality, CLIF-SOFA, SOFA, and Child-Pugh scores performed worse while MELD still performed well which was better than MELD-LA. The calibration curve of the MELD-LA score for 15-day mortality is presented in Figure 1(c) (*P* = 0.647). When stratified by causes of cirrhosis, as Figures 2 and 3 presented, MELD-LA gave the highest AUC for predicting 15-day mortality in cirrhotic patients caused by both alcohol and hepatitis or alcohol alone. While, as Figure 4 presented, the AUCs of SOFA and CLIF-SOFA scores were higher than other clinical scores for predicting 15-day mortality in cirrhotic patients caused by hepatitis alone. The performance of different clinical scores is showed in Tables 2 and 3 in detail.

## 4. Discussion

Our study evaluated for the first time the ability of MELD-LA to predict both short- and long-term mortality in critically ill cirrhotic patients stratified by causes of cirrhosis.

The research showed that cirrhotic patients admitted to ICU still had high mortality despite aggressive medical interventions, as has been reported before [3–5, 20]. Therefore, it is very important for risk assessment, optimal treatment selection, prolonging survival time, and improving survival quality, to have early, accurate, and objective predicting tools of mortality with accessible variables.

Recently, the MELD-LA score proved to be an early and objective predicting factor of in-hospital mortality in cirrhotic patients [9, 10]. We further investigated its value in predicting critically ill cirrhotic patients' short- and long-term prognosis, stratified by different etiologies. Our study confirmed that the MELD-LA score showed optimal discrimination value in predicting critically ill cirrhotic patients' short-term prognosis, especially caused by both alcohol and hepatitis or alcohol alone. However, for predicting long-term prognosis, MELD performed better. The prediction of critically ill cirrhotic patients' short-term mortality can be enhanced by lactate while the value of lactate in predicting long-term mortality requires further research [21]. MELD-LA score was related to lactate, may resulting in the ability of MELD-LA to predict long-term mortality worse than short-term mortality. Besides, further study is needed to carry out on the reasons for the poor value of MELD-LA in predicting short-term prognosis in critically ill cirrhotic patients caused by hepatitis alone as compared to patients due to other etiologies. Another clinically relevant study result is the statistically significant difference between survivors and nonsurvivors in terms of platelet count. This may be due to different bleeding risks caused by different platelet counts, while there are other studies that show that platelet count does not predict unprovoked major or minor bleeding in cirrhotic patients [22]. Thus, further research is needed.

Some potentially clinical applications of MELD-LA are described as follows. With lactate being associated with acute hepatic impairment [23–26] and used to assess the disease's severity in critically ill patients [27–31], the inclusion of lactate can more precisely show systemic lesions occurring in cirrhotic patients. MELD-LA score at admission can promptly and accurately assess the severity of cirrhosis and may be useful for stratifying patients that require higher levels of care or earlier interventions. Cirrhotic patients with an MELD-LA score >15 have extremely severe hepatic failure and higher short-term risk of death. An MELD-LA score >15 may indicate that the patient need liver transplantation. MELD-LA scores during hospitalization may help to identify patients that are not responding well to current treatment, which may allow for discussing treatment adjustment or palliative care earlier.

However, our research has limitations. This was a retrospective research conducted in a single institution. A future prospective multicentered study is needed. Besides, part of classic clinical scores were included, but others were excluded. In addition, the mortality was defined as all-cause mortality so it may be affected by other causes of death. Finally, therapeutic measures were not taken into account such as anticoagulant treatment, which may affect prognosis of patients. Anticoagulant treatment to treat portal vein thrombosis potentially improved the survival of patients with cirrhosis and such a complication [32]. We will conduct studies to solve these problems in the future.

## 5. Conclusions

The MELD-LA score, a recently-developed scoring system, has significantly superior performance in predicting short-term prognosis in critically ill cirrhotic patients, especially caused by both alcohol and hepatitis or alcohol alone. For predicting long-term prognosis, MELD performs better. Moreover, the MELD-LA score's potentially clinical applications need our further consideration and exploration.

## Figures and Tables

**Table 1:** Characteristics of the study population, stratified by survival.

Parameter	All patients (*N* = 469)	Survivors (*N* = 338)	Nonsurvivors (*N* = 131)	*P* value
Age (years)	55.6 (48.8–65.0)	55.7 (47.9–65.7)	55.1 (49.9–63.7)	0.804
Sex: male	307 (65.5)	223 (66.0)	84 (64.1)	0.705
BMI (kg/m^2^)	28.5 (24.1–32.4)	28.5 (24.0–32.3)	29.2 (25.1–33.5)	0.344
*Ethnicity*				0.159
White	342 (72.9)	251 (74.3)	91 (69.5)	
Black	30 (6.4)	24 (7.1)	6 (4.6)	
Others	97 (20.7)	63 (18.6)	34 (26.0)	
*Causes of cirrhosis*				
Alcoholic	260 (55.4)	182 (53.8)	78 (59.5)	0.266
Hepatitis B	12 (2.6)	7 (2.1)	5 (3.8)	0.283
Hepatitis C	118 (25.2)	81 (24.0)	37 (28.2)	0.338
Biliary	8 (1.7)	8 (2.4)	0 (0.0)	0.113
Autoimmune	4 (0.9)	3 (0.9)	1 (0.8)	1.000
Others	133 (28.4)	103 (30.5)	30 (22.9)	0.103
*Primary cause of ICU admission*				
Infection/sepsis	129 (27.5)	95 (28.1)	34 (26.0)	0.640
Bleeding	107 (22.8)	83 (24.6)	24 (18.3)	0.149
Respiratory	16 (3.4)	12 (3.6)	4 (3.1)	1.000
Cardiovascular	54 (11.5)	44 (13.0)	10 (7.6)	0.101
Renal failure	24 (5.1)	11 (3.3)	13 (9.9)	0.003
Neurological failure	49 (10.4)	36 (10.7)	13 (9.9)	0.817
Others	90 (19.2)	57 (16.9)	33 (25.2)	0.040
*Comorbidity*				
Hypertension	117 (24.9)	82 (24.3)	35 (26.7)	0.581
Diabetes	121 (25.8)	85 (25.1)	36 (27.5)	0.604
*Vital signs*				
Temperature (°C)	36.7 (36.3–37.2)	36.7 (36.4–37.3)	36.4 (36.0–36.9)	<0.001
Heart rate	90.5 (79.0–103.3)	88.6 (78.0–102.2)	95.1 (81.3–104.4)	0.046
MAP (mmHg)	73.0 (67.6–80.7)	74.8 (69.1–82.2)	69.6 (64.3–75.1)	<0.001
Respiratory rate	18.9 (16.1–22.1)	18.4 (15.9–21.0)	20.5 (17.1–24.7)	<0.001
SpO_2_/FiO_2_	183.0 (172.9–456.6)	198.7 (175.1–457.7)	174.9 (168.8–454.5)	<0.001
24-h urine output (mL)	1119 (570–1890)	1325 (759–2000)	571 (175–1119)	<0.001
Mechanical ventilation duration (hours)	37.0 (0.0–121.7)	22.4 (0.0–123.0)	59.7 (12.0–120.5)	0.006
Length of ICU stay (day)	4.0 (2.4–8.4)	3.9 (2.3–8.8)	4.2 (2.6–7.3)	0.571
*Laboratory parameters*				
Hb (mg/dL)	9.9 (9.0–11.1)	10.0 (9.1–11.2)	9.7 (8.6–11.0)	0.041
WBC (10^9^/L)	10.5 (7.4–15.7)	10.2 (7.4–14.6)	11.7 (7.5–19.3)	0.018
Platelet (10^9^/L)	99.8 (70.1–151.3)	108.2 (74.3–159.0)	86.0 (61.8–116.0)	<0.001
INR	1.7 (1.5–2.1)	1.6 (1.4–1.9)	2.1 (1.8–2.9)	<0.001
PT (seconds)	18.0 (15.6–21.4)	16.8 (15.3–19.7)	21.1 (18.4–25.2)	<0.001
PTT (seconds)	39.5 (33.5–49.2)	37.4 (32.4–44.3)	46.6 (39.3–63.2)	<0.001
Glucose (mg/dL)	126.7 (103.0–158.0)	127.5 (103.3–157.6)	124.5 (101.2–158.3)	0.823
Sodium (mEq/L)	138.0 (134.0–141.1)	138.0 (135.3–141.2)	136.0 (131.0–141.0)	<0.001
Potassium (mEq/L)	4.1 (3.7–4.5)	4.1 (3.7–4.4)	4.3 (3.8–4.7)	0.002
BUN (mg/dL)	29.5 (17.0–50.0)	26.0 (15.5–44.3)	41.4 (26.5–63.0)	<0.001
Creatinine (mg/dL)	1.3 (0.8–2.5)	1.1 (0.8–2.0)	1.9 (1.2–3.4)	<0.001
Bilirubin (mg/dL)	3.3 (1.6–7.7)	2.8 (1.4–5.3)	7.2 (3.2–16.6)	<0.001
Albumin (g/dL)	2.8 (2.6–3.0)	2.8 (2.6–3.0)	2.8 (2.5–3.1)	0.291
Lactate (mmol/L)	2.2 (1.6–3.8)	2.0 (1.5–3.0)	3.4 (2.1–6.6)	<0.001
*Clinical scores*				
Child-Pugh	10 (9–11)	10 (9–11)	11 (10–11)	<0.001
SOFA	9 (6–11)	8 (6–10)	12 (10–14)	<0.001
CLIF-SOFA	9 (7–12)	9 (6–11)	12 (10–15)	<0.001
MELD	16 (10–25)	13 (8–20)	26 (17–34)	<0.001
MELD-LA	15 (11–18)	13 (10–16)	19 (16–24)	<0.001

Values are expressed as *n* (%) or median (IQR). UC, ulcerative colitis; CD, Crohn' s disease; BMI, body mass index.

**Table 2:** Model discrimination for mortality in the whole study population.

Mortality	15-day mortality				5-year mortality			
Prognostic models	AUROC (95% CI)	Cut-off point	Sensitivity	Specificity	AUROC (95% CI)	Cut-off point	Sensitivity	Specificity
Child-Pugh	0.611 (0.558–0.665)	11	0.63	0.56	0.575 (0.523–0.627)	10	0.74	0.38
SOFA	0.802 (0.757–0.846)	10	0.78	0.71	0.669 (0.620–0.719)	10	0.52	0.72
CLIF-SOFA	0.794 (0.748–0.840)	11	0.74	0.75	0.684 (0.636–0.732)	10	0.62	0.69
MELD	0.775 (0.728–0.822)	20	0.69	0.74	0.721 (0.674–0.767)	17	0.63	0.73
MELD-LA	0.808 (0.765–0.852)	15	0.81	0.65	0.713 (0.666–0.760)	14	0.70	0.64

DeLong test was used to compare the AUC between MELD-LA and other clinical models. AUROC, area under the receiver operating characteristic curve; CI, confidence interval; SOFA, sequential organ failure assessment; CLIF-SOFA, chronic liver failure-sequential organ failure assessment; MELD, model for end-stage liver disease; MELD-LA, model for end-stage liver disease-lactate.

**Table 3:** Model discrimination for mortality, stratified by causes of cirrhosis.

AUROC (95% CI)						
Causes of cirrhosis	Alcoholic and hepatitis (*n* = 59)		Alcoholic (*n* = 201)		Hepatitis (*n* = 64)	
Mortality	15-day mortality	5-year mortality	15-day mortality	5-year mortality	15-day mortality	5-year mortality
Child-Pugh	0.592 (0.438–0.747)	0.707 (0.571–0.842)	0.563 (0.483–0.642)	0.542 (0.461–0.623)	0.505 (0.354–0.655)	0.548 (0.396–0.700)
SOFA	0.735 (0.583–0.887)	0.666 (0.525–0.807)	0.799 (0.734–0.863)	0.681 (0.606–0.756)	0.818 (0.711–0.926)	0.751 (0.632–0.870)
CLIF-SOFA	0.723 (0.575–0.872)	0.672 (0.532–0.813)	0.781 (0.713–0.850)	0.666 (0.590–0.742)	0.801 (0.679–0.924)	0.756 (0.635–0.877)
MELD	0.755 (0.619–0.891)	0.749 (0.617–0.881)	0.787 (0.720–0.853)	0.721 (0.649–0.794)	0.714 (0.578–0.851)	0.750 (0.627–0.873)
MELD-LA	0.783 (0.651–0.915)	0.678 (0.535–0.820)	0.805 (0.743–0.867)	0.732 (0.660–0.803)	0.762 (0.627–0.897)	0.750 (0.628–0.872)

DeLong test was used to compare the AUC between MELD-LA and other clinical models. AUROC, area under the receiver operating characteristic curve; CI, confidence interval; SOFA, sequential organ failure assessment; CLIF-SOFA, chronic liver failure-sequential organ failure assessment; MELD, model for end-stage liver disease; MELD-LA, model for end-stage liver disease-lactate.

## Data Availability

The data used to support the findings of this study are available from the author upon request.
